# A Network Approach for the Accurate Characterization of Water Lines Observable in Astronomical Masers and Extragalactic Environments

**DOI:** 10.1021/acsearthspacechem.4c00161

**Published:** 2024-08-09

**Authors:** Wim Ubachs, Attila G. Császár, Meissa L. Diouf, Frank M. J. Cozijn, Roland Tóbiás

**Affiliations:** † Department of Physics and Astronomy, LaserLaB, Vrije Universiteit, De Boelelaan 1081, 1081 HV Amsterdam, The Netherlands; ‡ Institute of Chemistry, ELTE Eötvös Loránd University, H-1518 Budapest 112, P.O. Box 32, Hungary; § HUN-REN−ELTE Complex Chemical Systems Research Group, H-1117 Budapest, Pázmány Péter sétány 1/A, Hungary

**Keywords:** water, spectroscopy, radio frequencies, masers, extragalactic environments, Lamb dips

## Abstract

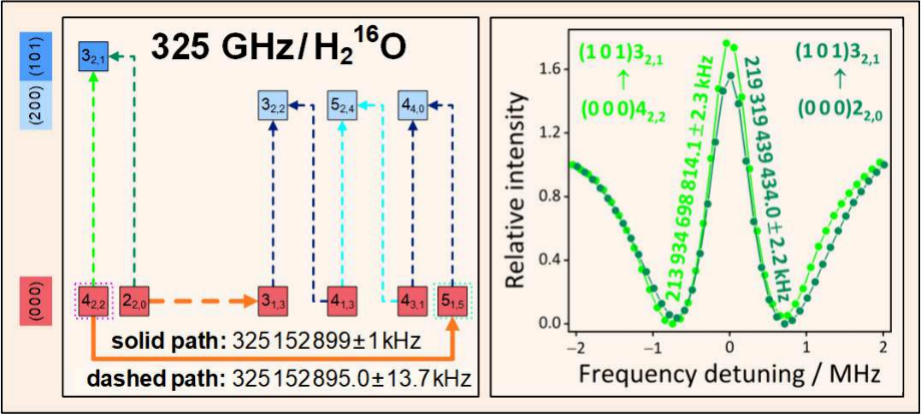

The water molecule, crucial to the chemical composition and dynamics of the universe, is typically identified in its gas phase via radio and submillimeter transitions, with frequencies up to a few THz. To understand the physicochemical behavior of astronomical objects, accurate transition frequencies are required for these lines. From a set of 26 new and 564 previous Lamb dip measurements, utilizing our ultrasensitive laser-based spectrometers in the near-infrared region, ultrahigh-precision spectroscopic networks were set up for H_2_^16^O and H_2_^18^O, augmented with 40 extremely accurate frequencies taken from the literature. Based on kHz-accuracy paths of these networks, considerably improved line-center frequencies have been obtained for 35 observed or predicted maser lines of H_2_^16^O, as well as for 14 transitions of astronomical significance of H_2_^18^O. These reference frequencies, attached with 5–25 kHz uncertainties, may help future studies in various fields of astrochemistry and astrophysics, in particular when precise information is demanded about Doppler-velocity components, including the gas flows of galactic cores, the kinematics of planetary nebulae, or the motion in exoplanetary atmospheres.

## 1 Introduction

Water is a key molecular ingredient of the chemical universe, ubiquitous on Earth, in our planetary system, in interstellar clouds inside our Milky way, and in far-distant galaxies. The large dipole moment of the water molecule supports its effective cooling in the interstellar medium, playing a fundamental role in the physical development of galaxies, as well as in the formation of planetary systems.^1^ Furthermore, water acts as a key player in the evolution of life on Earth and hence it is a target species for the spectroscopic investigation of exoplanets.

Besides the gas phase, water also exists in an amorphous solid phase, where the diverse forms of water-based ices exhibit characteristic spectral features.^2−4^ The surface of these water-based ices facilitates the production of several interstellar components, ranging from small species^5^ to complex organic molecules.^6^ The chemistry involving water ices, irradiated by ultraviolet radiation, was extensively investigated by Harold Linnartz, to whom this article is dedicated.

The presence, the amount, and the distribution of water molecules can be studied via spectroscopic means. A wide range of astronomical objects and phenomena can be explored by measuring water transitions in the gas phase, either in emission or in absorption, which fall into the radio frequency domain. Radio astronomy from Earth-bound observatories focuses on line frequencies below 1 THz, representing an atmospheric window. For low-altitude radio telescopes, the observation range is limited to below 100 GHz.

The first identification of water in the interstellar medium, in fact through a maser transition around 22 GHz, was established at the Hat Creek Observatory.^7^ A decade later,^8^ high-velocity molecular outflows were found in Orion-KL by probing this 22 GHz line via interferometric radio astronomy. In 1993, the Nobeyama radio station discovered water maser emission of extreme velocity in a distant galaxy.^9^ The near-to-sea-level Effelsberg 100-m telescope allowed the study of water in the early Universe at redshift *z* = 2.64 or *f* = 6 GHz.^10^ The improved sensitivity of the Atacama Large Millimeter Array (ALMA), positioned at an altitude above 5 km, enabled the investigation of water lines at *z* = 6.9 or *f* = 3 GHz.^11^ ALMA made it possible to scrutinize the role of water in planet formation via water masers at 183, 321, and 658 GHz.^12,13^ The ALMA observatory, currently the most sensitive radio telescope in the mm and sub-mm regions, aims at quadrupling the system bandwidths in its ALMA 2030 project,^14^ further improving its spectral resolution.

For frequencies above 1 THz, satellite-based observations are required, due to the opacity of water in the Earth’s atmosphere. Utilization of the HIFI (Heterodyne Instrument for the Far Infrared) device aboard the Herschel observatory ensured the detection of rotational water lines at 540–1700 GHz, used for the analysis of the physicochemical conditions in the water emitting region toward the high-mass protostar AFGL 2591.^15^ At even higher frequencies, water absorption lines were identified around 37 THz with the Spitzer telescope, finding a large amount of water in the atmosphere of a transiting exoplanet.^16^ Using the 120–500 THz range of the James Webb Space Telescope (JWST), water could also be detected in hot exoplanetary atmospheres.^17^ Water lines in the visible range (585–600 nm) were also employed to study the Earth’s atmosphere.^18^

With the advent of outer-atmospheric spectral devices, such as the (by now inactive) Herschel and SOFIA (Stratospheric Observatory for Infrared Astronomy) instruments, the need for refined line frequencies in the sub-mm region has been stressed in advanced astronomical investigations.^19,20^ This equally holds for ALMA, which covers a wide frequency range below 1 THz (more specifically, 35–950 GHz). Hence, it is an important task to increase the accuracy of water lines applied in radio and sub-mm astronomy. A list of maser transitions detectable below 2 THz has been been recently compiled by Gray et al. for H_2_^16^O,^21^ helping future astrophysical applications. In addition, two studies^22,23^ reported a couple of H_2_^18^O lines observed via the PACS (Photoconductor Array Camera and Spectrometer) and HIFI instruments on Herschel.

The principal goal of this work is to show how the spectroscopic-network-assisted precision spectroscopy (SNAPS) approach^24^ can be applied to deduce accurate line positions for astronomically relevant transitions of two water isotopologues H_2_^16^O and H_2_^18^O. The present SNAPS analysis is based on ultraprecise experimental results of previous studies,^24−32^ along with 26 new Lamb dips measured during this study. The astronomical examples guiding our discussion include 48 H_2_^16^O maser lines from ref (21) and (14) H_2_^18^O lines from refs (22), (23).

## 2 SNAPS-Based Line Selection

As advocated in our previous studies,^24,30−32^ the SNAPS protocol is a particularly useful tool when the aim is to extract the maximal amount of accurate spectroscopic information from a limited number of precision-spectroscopy experiments. SNAPS facilitates the selection of connected transition sequences (*t*_1_, *t*_2_, ..., *t*_*N*_), where *t*_*i*_ is incident to the (*s*_*i*_, *s*_*i*+1_) state pair and the intermediate (*s*_2_, *s*_3_, ..., *s*_*N*_) states are pairwise distinct. A connected transition sequence may be a path/cycle, depending on whether the exterior (*s*_1_ and *s*_*N*+1_) states are distinct/identical. A path can be applied to obtain an accurate energy difference between its starting (*s*_1_) and ending (*s*_*N*+1_) state, whereas a cycle helps to confirm the internal accuracy of its underlying transitions through the analysis of its discrepancy.^33,34^ For further details on SNAPS, see refs (24) and (32).

The SNAPS scheme has been used to derive accurate rovibrational energies for H_2_^16^O^24,31,32^^24,31,32^ and H_2_^18^O,^30,32^ within the ground and highly excited vibrational states. These accurate results relied on more than 500 Lamb dips detected, with 1.5–38.9 kHz accuracy, via two NICE–OHMS (noise-immune cavity-enhanced optical-heterodyne molecular spectroscopy) setups.^35,36^ For both species, the lowest *ortho* energies, which cannot be extracted purely from experiments due to the lack of observed *ortho* ↔ *para* lines,^37^ could be deduced with 6–8 kHz uncertainty. These two energies were taken from effective Hamiltonian (EH) fits, as well as from network paths where the *ortho* and *para* subpaths were concatenated with (exceedingly small) accurate, first-principles *ortho*–*para* splittings as special links.^24,30^ In the following section, the sets of high-precision H_2_^16^O and H_2_^18^O lines are reviewed and extended with additional Lamb-dip measurements, yielding kHz-accuracy predictions for astronomically important water lines.

In what follows, the H_2_^16^O and H_2_^18^O energy levels are represented with , where *v*_1_, *v*_2_, and *v*_3_ are the normal-mode vibrational quantum numbers of the symmetric stretch, bend, and asymmetric stretch motions, respectively, *J* is the overall rotational quantum number, whereas *K*_*a*_ and *K*_*c*_ are the conventional prolate- and oblate-top rotational quantum numbers, respectively. For a specific state, (a) the labels *ortho*/*para* and *even*/*odd* correspond to (−1)^*v*_3_+*K*_*a*_+*K*_*c*_^= +1/–1 and (−1)^*K*_*c*_^= +1/–1, respectively, and (b) the polyad number is given as *P* = 2*v*_1_ + *v*_2_ + 2*v*_3_. Moreover,  designates a rovibrational line, where ′ and ^*″*^ distinguish between its upper and lower states, respectively.^38^ Unless otherwise noted, the words “transition” and “line” indicate a one-photon, dipole-allowed, rovibrational transition measured under absorption conditions.

## 3 The Ultraprecise H_2_^16^O and H_2_^18^O Networks

As part of the SNAPS procedure, ultraprecise spectroscopic networks were formed for H_2_^16^O and H_2_^18^O, involving NICE–OHMS transitions at 1.2 and 1.4 μm wavelengths, augmented with a few extremely accurate lines collected from the literature.^25−29,39^ To ensure connectivity among (0 0 0) states within the *ortho*-H_2_^X^ O and *para*-H_2_^X^ O subnetworks (X = 16, 18), 0 ⇐ 0 and 4 ⇐ 0 lines have been concatenated, where *P*′ ⇐ *P*″ denotes a transition between polyads *P*′ and *P*″. The utilization of a few 0 ⇐ 0 lines, taken from ultrahigh-precision microwave measurements,^25−27^ is required, because the subsets of *even*- and *odd*-parity (0 0 0) states cannot be linked with near-infrared dipole transitions. Note that (0 1 0) states are also included in the H_2_^16^O network, whose *ortho*/*para* subnetworks become connected via highly accurate 5 ⇐ 1 and 5 ⇐ 0 lines. In the remainder of this section, a brief description is given about the data sources which were employed during the compilation of the (hyperfine-free) ultraprecise H_2_^16^O and H_2_^18^O networks.

In an early beam-maser study by Kukolich,^25^ the hyperfine and Zeeman structure of the 22 GHz maser line was probed with 50 Hz accuracy, yielding so far the most accurate line center for water. Later, Golubiatnikov et al.^26^ analyzed the spectrum of water in the 180–560 GHz frequency region, identifying 13 and 6 Lamb-dip transitions, with 1–20 kHz accuracy, for H_2_^16^O and H_2_^18^O, respectively. In the case of the *ortho* transitions, some well-separated hyperfine components could also be resolved.^26^ Cazzoli et al.^27^ conducted an analysis for seven *ortho*-H_2_^16^O lines, via an ultrahigh-resolution spectrometer in the 320–620 GHz range. For the seven hyperfine-free rotational lines, derived from the hyperfine components, sub-kHz accuracy could be attained. In the near-infrared region, three papers^28,29,39^ reported Lamb dips for H_2_^16^O with a few kHz uncertainty^28,29^ or even better,^39^ measured with the cavity ring-down spectroscopy (CRDS) technique^40^ in saturation. These near-infrared lines do not participate in sufficiently accurate Λ schemes (i.e., pairs of transitions sharing the same upper state) in the H_2_^16^O network, preventing their use in the derivation of 0 ⇐ 0 line frequencies.

Taking advantage of the SNAPS approach and the ultrasensitive NICE–OHMS technique, a set of near-infrared Lamb-dip transitions was measured for H_2_^16^O and H_2_^18^O, leading to 1.5–10 kHz accuracy for the strongest lines.^24,30−32^ In ref (24), 156 carefully chosen transitions were accurately measured, which produced kHz-accuracy absolute energies for all but two (0 0 0) rotational states up to *J* = 8. Later,^30^ 195 lines were detected for H_2_^18^O under saturation and then were used to derive empirical energies for all  states. Subsequently,^31^ the SNAPS method was employed to deduce rovibrational energies for the  energy levels, as well, after the inclusion of 71 additional Lamb-dip lines in the ultraprecise H_2_^16^O network. In a very recent study,^32^ the focus was on the hubs (i.e., states incident to the largest number of observed transitions) within the experimental H_2_^16^O and H_2_^18^O networks assembled in ref (41). For 183 hubs, lying on the (0 1 0) vibrational state of H_2_^16^O and the (0 0 0) state of both species, rovibrational energies were determined with kHz-level uncertainties,^32^ involving 135/8 new Lamb dips for H_2_^16^O/H_2_^18^O. The four experimental studies mentioned in this paragraph provide the major part of the lines in the ultraprecise H_2_^16^O and H_2_^18^O networks.

**Figure 1:**
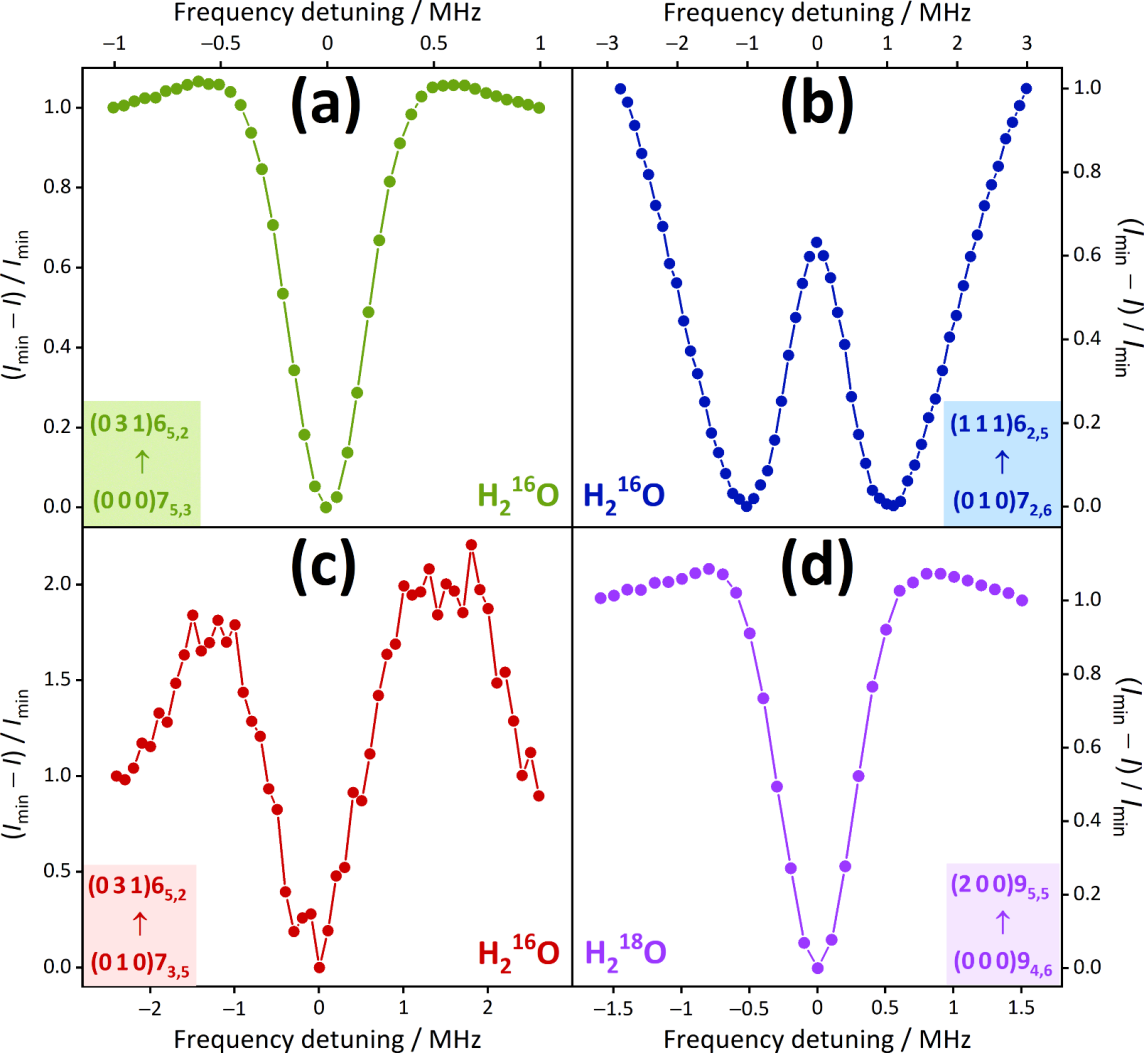
Typical Lamb dips detected during this study for H_2_^16^O and H_2_^18^O. Panels (a) and (d) present two regular Lamb-dip profiles, characterized by a single dip. Panel (b) displays an inverted (double-dip) profile,^31^ which occurs for transitions with large (>0.5 s^–1^) Einstein-*A* coefficients. Panel (c) exhibits an H_2_^16^O line with very low (9.7 × 10^–28^ molecule^–1^) intensity, obtained via averaging over 20 scans. To facilitate their visual comparison, these spectra are mapped onto the (*I*_min_ – *I*)/*I*_min_ relative intensity scale, where *I* means the intensity at a detuning point for a specific transition, and *I*_min_ is the lowest intensity in the ±3 MHz detuning region.

As a minor but important extension, 14/12 NICE–OHMS transitions are reported here for H_2_^16^O/H_2_^18^O, leading to altogether 411/220 lines in the ultrahigh-accuracy H_2_^16^O/H_2_^18^O network assembled during this study. All the new transitions, listed in Table 1, were observed with our newer NICE–OHMS setup,^36^ to reach as low frequency uncertainties as feasible. The new H_2_^16^O transitions serve as the basis for a refined determination of the maser frequencies taken from Gray et al.^21^ (see Sec. 4), while those measured for H_2_^18^O are used to improve the frequencies of some less accurate 0 ⇐ 0 lines in the full experimental H_2_^18^O network.^41^ For the newly recorded H_2_^16^O and H_2_^18^O lines, the 1σ uncertainties were obtained via
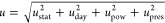
1where *u*_stat_, *u*_day_, *u*_pow_, and *u*_pres_ are the statistical, day-to-day, power-shift, and pressure-shift uncertainties, respectively. Four typical recordings, yielding regular and inverted^31^ Lamb-dip profiles, are plotted in Figure 1.

**Table 1:** : List of New Lamb-Dip Lines Recorded for H_2_^16^O and H_2_^18^O

	NICE–OHMS (this work)^a^			Doppler-limited measurements^c^		
Species	Line frequency/kHz	*p*/Pa	Rovibrational assignment^b^	Dev./MHz	Unc./MHz	Ref.
H_2_^16^O	210 761 755 868.8 ± 51.2^d^^,^^e^	0.55	(0 3 1)6_5,2_ ← (0 1 0)7_3,5_	—	—	—
	210 826 297 225.3 ± 6.7^f^	0.25	(1 1 1)6_2,5_ ← (0 1 0)7_2,6_	30.0	3.0	(42)
	211 120 145 828.8 ± 3.0	0.10	(2 0 0)5_4,2_ ← (0 0 0)5_5,1_	47.3	3.0	(42)
	213 934 698 814.1 ± 2.3^f^	0.05	(1 0 1)3_2,1_ ← (0 0 0)4_2,2_	–3.6	3.0	(43)
	214 323 335 908.9 ± 7.0^f^	0.25	(1 1 1)7_5,3_ ← (0 1 0)7_5,2_	–207.5	3.0	(42)
	218 258 351 906.2 ± 2.7^f^	0.02	(2 0 0)5_4,2_ ← (0 0 0)5_3,3_	–0.5	3.0	(42)
	219 063 350 000.9 ± 1.8^f^	0.01	(2 0 0)6_6,0_ ← (0 0 0)6_5,1_	3.5	3.0	(42)
	219 319 439 434.0 ± 2.2^f^	0.02	(1 0 1)3_2,1_ ← (0 0 0)2_2,0_	–1.7	3.0	(44)
	219 651 805 235.8 ± 5.4^f^	0.25	(1 1 1)6_2,5_ ← (0 1 0)5_2,4_	65.7	3.0	(42)
	249 181 073 419.9 ± 10.9^e^^,^^g^	0.50	(1 1 1)6_5,2_ ← (0 0 0)7_7,1_	140.0	30.0	(45)
	250 114 135 377.1 ± 2.5	0.09	(0 3 1)6_4,2_ ← (0 0 0)7_4,3_	0.4	5.1	(46)
	251 242 164 156.9 ± 2.5	0.09	(1 1 1)7_2,6_ ← (0 0 0)8_4,5_	3.7	4.8	(46)
	251 386 571 392.8 ± 2.4	0.09	(0 3 1)7_5,3_ ← (0 0 0)8_5,4_	2.0	5.4	(46)
	252 142 342 842.5 ± 2.6	0.08	(0 3 1)6_5,2_ ← (0 0 0)7_5,3_	–1.4	3.9	(46)
H_2_^18^O	217 879 222 532.8 ± 3.7^f^	0.07	(1 0 1)9_3,6_ ← (0 0 0)9_3,7_	–3.4	3.0	(42)
	214 778 899 384.2 ± 3.7^d^	0.07	(2 0 0)9_5,5_ ← (0 0 0)10_2,8_	—	—	—
	217 733 343 682.5 ± 4.2	0.12	(2 0 0)9_5,5_ ← (0 0 0)9_4,6_	1.6	3.0	(42)
	218 129 328 936.8 ± 4.9	0.12	(2 0 0)8_3,5_ ← (0 0 0)7_4,4_	–10.0	30.0	(47)
	213 512 114 337.3 ± 4.2	0.12	(2 0 0)8_3,5_ ← (0 0 0)9_2,8_	9.8	30.0	(47)
	219 557 374 515.1 ± 4.2	0.12	(0 0 2)8_0,8_ ← (0 0 0)7_3,5_	9.1	15.0	(48)
	216 453 568 294.1 ± 4.2	0.12	(0 0 2)8_0,8_ ← (0 0 0)9_1,9_	–11.0	15.0	(48)
	216 715 103 815.8 ± 4.2	0.12	(0 0 2)10_1,9_ ← (0 0 0)10_2,8_	–18.9	30.0	(47)
	214 144 113 987.0 ± 4.9	0.12	(0 0 2)10_1,9_ ← (0 0 0)11_2,10_	120.4	30.0	(47)
	215 950 444 612.3 ± 5.8^d^	0.12	(2 0 0)9_6,4_ ← (0 0 0)10_3,7_	—	—	—
	215 518 142 895.5 ± 2.9	0.08	(1 0 1)9_5,5_ ← (0 0 0)10_3,8_	94.9	15.0	(48)
	217 886 018 267.3 ± 19.7^d^^,^^e^	0.24	(0 0 2)8_7,1_ ← (0 0 0)9_6,4_	—	—	—

a Room-temperature Lamb-dip positions, with associated 1σ uncertainties, at pressure values given in the third column.

b Rovibrational assignments are given as  (see Sec. 2).

c The most accurate Doppler-broadened experimental results are taken from the literature (see column “Ref.”). The column “Dev.” lists the deviations of the Doppler-limited positions from their Lamb-dip counterparts. The column “Unc.” contains uncertainty estimates provided in the references. Except ref (46), these sources report only average uncertainties for the observed lines (thus, it is not surprising that some of the respective deviations grow above 4σ).

d Transitions not measured via Doppler spectroscopy.

e Lines, three in total, characterized by very low (<10^–26^ cm molecule^–1^) absorption intensities.

f Transitions, eight in total, with inverted Lamb-dip profiles.^31^

g Line forming part of an unresolved *ortho*–*para* doublet in Doppler-limited spectra at room temperature.

As apparent from Table 1, most of the new line centers could be retrieved with an accuracy of 2–52 kHz at a total pressure of 0.01–0.55 Pa. Taking an effective pressure-shift coefficient, 20 kHz Pa^–1^,^24,30^ into account, a pressure-shift uncertainty of 0.2–11 kHz is included in the uncertainty budget. The three less accurate lines with >10 kHz uncertainties are characterized by small (<10^–26^ molecule^–1^) intensities, leading to somewhat lower signal-to-noise ratios. A low-intensity H_2_^18^O transition, with 20 kHz accuracy, was detected at 217 886 018 263.8 ± 25.3 kHz^30^ with our older setup,^35^ exhibiting only a negligible redshift of 3.5 kHz from the new line-center position.

Table 1 also provides a comparison between the new Lamb-dip lines and previous Doppler-limited spectroscopic results.^42−44,46−48^ This comparison reveals significant shifts, exceeding 4σ, for six Doppler-broadened observations, while the rest of the former frequencies agree within 2σ with the NICE–OHMS values. Overall, the NICE–OHMS measurements yield a considerable improvement for the 26 line frequencies, corresponding to 3 orders of magnitude, when compared to their previous determinations.^42−44,46−48^

## 4 Extraction of Frequency Predictions from Network Paths

**Figure 2:**
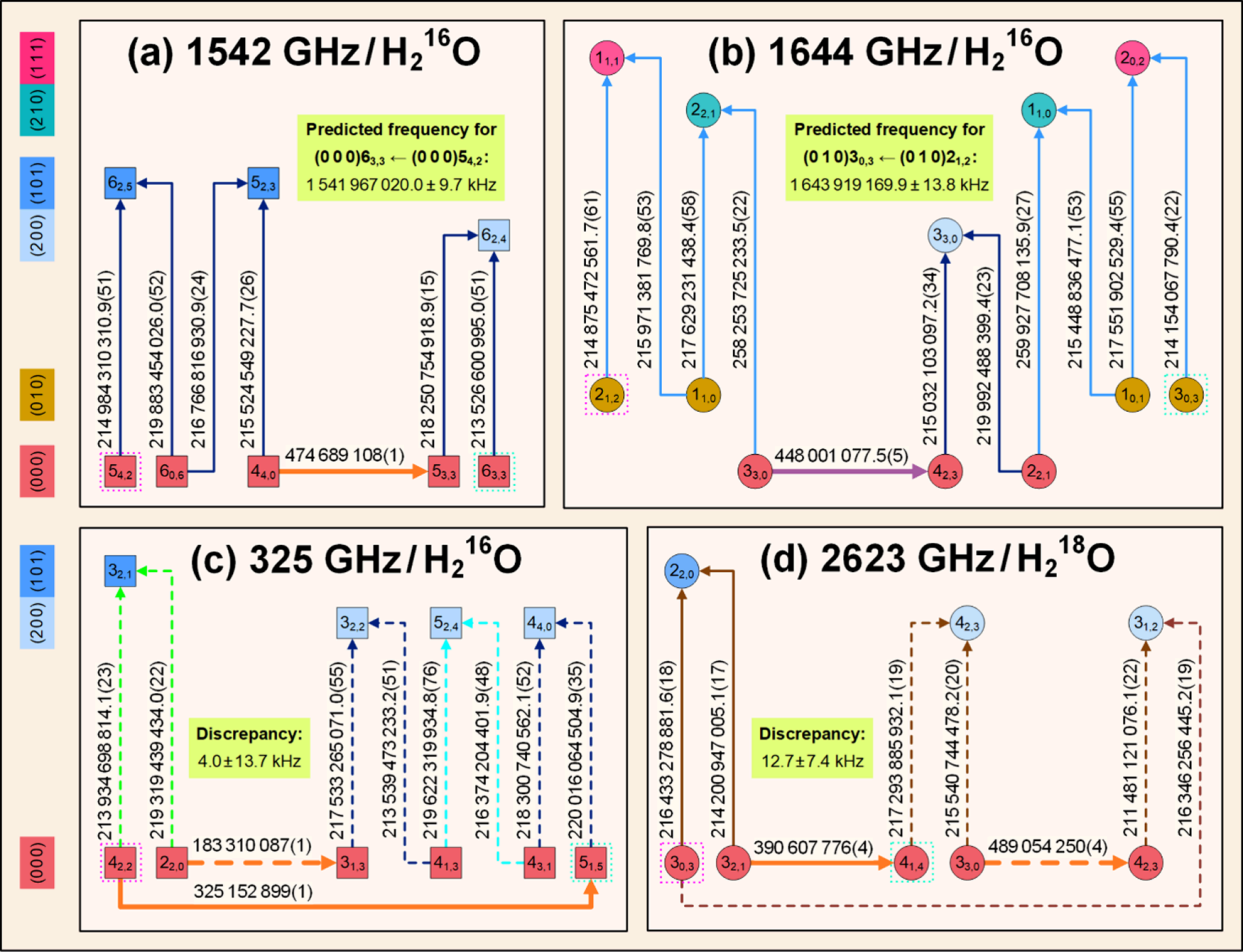
Typical short paths and cycles used for the characterization of astronomical H_2_^16^O and H_2_^18^O lines. The *ortho* and *para* states of this figure are symbolized with circles and squares, respectively. For these states, the  labels are written out explicitly, whereas the (*v*_1_*v*_2_*v*_3_) triplets are shown in the left-side color legend. The green arrows illustrate new Lamb-dip transitions, while those with dark blue, orange, purple, brown, cyan, and light blue colors are ultrahigh-accuracy lines taken from refs (24, 26, 27, 30, 31), and (32), respectively. For the pure rotational transitions included on the paths, thicker arrows are used. The numbers on the arrows designate frequencies in kHz, with 1σ uncertainties of the last digits in parentheses. The solid arrows constitute the “best” (lowest-uncertainty) paths between their starting and ending states, distinguished with dotted magenta and mint boxes, respectively. The dashed arrows form alternative paths, producing cycles with the solid ones. The approximate positions, related to transitions between the starting and ending states of the best paths, are shown at the top of the panels. The yellowish-green boxes provide predicted frequencies and discrepancies, with their 1σ uncertainties, for paths [panels a and b] and cycles [panels c and d], respectively. For further details, see the text.

To derive a prediction for a transition frequency within the SNAPS approach, it is necessary to establish an uninterrupted connection between the upper and lower states of the predicted line. This connection must be secured by a path, whose starting and ending states correspond to the upper and lower states of the desired transition, respectively. In the ultraprecise H_2_^16^O and H_2_^18^O networks, such paths mostly involve sequential Λ schemes, ensuring a kind of “spectroscopic triangulation” via up and down jumps between polyads. Among certain Λ schemes, pure rotational transitions must also be inserted on a path, producing seamless connection between opposite-parity lower states. If there are multiple (line-disjoint) paths between the same starting and ending states, they represent independent predictions for the same transition, warranting a comparison among the alternative frequencies and their uncertainties. Two paths form one or more cycles, depending on whether they have two ore more common states, respectively. A few characteristic paths and cycles, employed during the determination of accurate frequencies for astronomically important H_2_^16^O and H_2_^18^O lines, are visualized in Figure 2, guiding our analysis in the remaining part of this section.

To understand how a frequency prediction can be obtained from a path, one must use the Ritz principle^49^ in a successive way.^24,32^ This process yields the following expression for the predicted frequency:
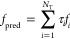
2whereby *N*_T_ is the number of transitions in the network, and *f*_*i*_ is the experimental frequency of the *i*th line preceded by a path-dependent “ternary” parameter, τ_*i*_. If the *i*th transition does not participate in this path, then τ_*i*_ = 0, otherwise τ_*i*_ is +1 or −1, depending on whether it points toward the upper or the lower state of the predicted line, respectively. For instance, the lines of Figure 2a have the following signs (from left to right): +1, −1, +1, −1, +1, +1, and −1. Supposing uncorrelated experimental errors, a well-defined 1σ uncertainty estimate can be formulated for *f*_pred_:
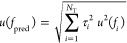
3where *u*(*f*_*i*_) is the 1σ uncertainty of the *f*_*i*_ frequency. For two independent predictions, *f*_pred_^I^ and *f*_pred_^II^, their discrepancy and its uncertainty, respectively, can be calculated as
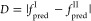
4and
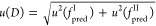
5If *D* ≤ 2*u*(*D*), then the two predictions are statistically identical at the 95% significance level.

**Figure 3:**
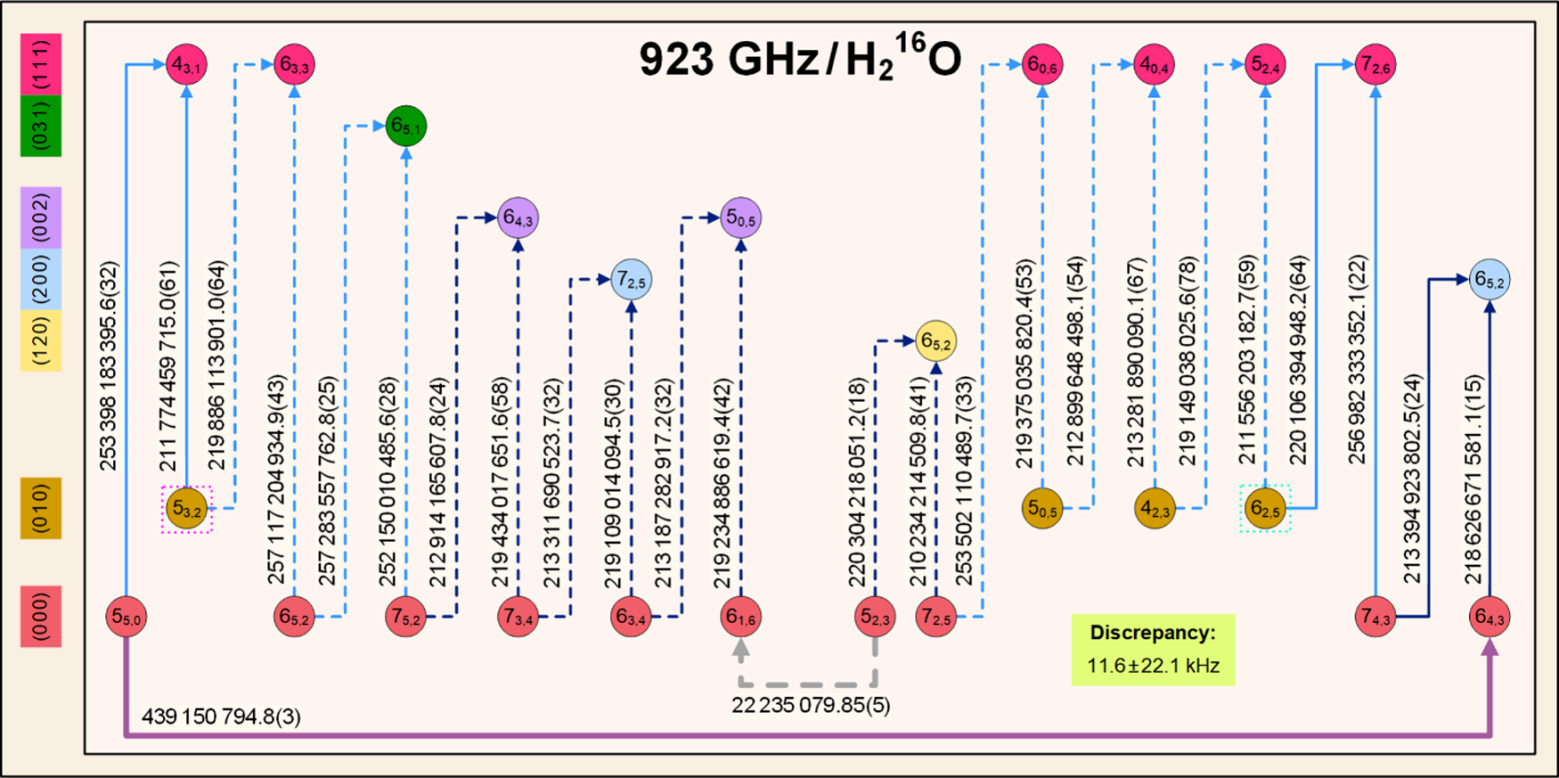
Example for a long cycle formed by two line-independent paths between two (0 1 0) states. The notation of this figure is the same as in Figure 2, with the extension that the gray arrow denotes a transition taken from ref (25).

To minimize the *u*(*f*_pred_) uncertainty, one must find a shortest path, called here a best path, between the upper and lower states of the predicted line within the ultraprecise H_2_^16^O/H_2_^18^O network. For this purpose, the Dijkstra algorithm^50^ can be invoked, using the *u*^2^(*f*_*i*_) values as edge weights. With the aid of best paths, one can bypass less accurate transitions, like the noisy transition of Figure 1c with 51.2 kHz uncertainty. Some specific examples for best paths are denoted with solid arrows in Figure 2, accompanied by alternative (dashed) paths in its last two panels. As obvious from Figures 2c and 2d, the best and the alternative predictions for the two 0 ⇐ 0 line frequencies agree well with each other, exhibiting discrepancies within the 2σ limit. Similarly good agreement is seen for a 1 ⇐ 1 line, (0 1 0)6_2,5_ ← (0 1 0)5_3,2_, expressed with two long paths in Figure 3.

## 5 Improved Frequencies for Astronomical Water Lines

Built upon the best paths taken from the ultrahigh-accuracy H_2_^16^O/H_2_^18^O networks, accurate frequencies have been determined, with definitive uncertainties, for selected astronomical transitions of H_2_^16^O and H_2_^18^O. From the large number of water lines relevant for radio astronomy, a small, but representative collection has been compiled, based upon ref (21) for H_2_^16^O, as well as refs (22) and (23) for H_2_^18^O. For all transitions of this collection, the recommended frequencies, extracted from the best paths, are correlated with those of the most precise laboratory experiments.^25−27,51−63^ The best paths yielding the recommended frequencies, augmented with a line-by-line comparison to multiple experimental positions existing for the same astronomical line, are provided as Supporting Information. These comparison files also contain SNAPS values derived without using the new lines of Table 1, showing full agreement between the two kinds of SNAPS predictions.

### 5.1 H_2_^16^O Lines

In a study by Gray et al.,^21^ a list of observed and predicted H_2_^16^O maser transitions was composed in the 0–1910 GHz frequency range, most of which play an essential role in the radiative-transfer models of various astrophysical environments. From that list, an excerpt was made, see Table 2, covering all the lines for which SNAPS-predicted frequencies are available. This excerpt does not include transitions pertaining to the *P* = 2 polyad, nor those with large *J* or *K*_*a*_ values, as they are not accessible from the ultraprecise H_2_^16^O network.

**Table 2:** Recommended Frequencies for H_2_^16^O Maser Lines Collected from Ref (21)

Line frequency			Laboratory measurements^c^			
Rest^a^/GHz	Recommended^b^/kHz	Rovibrational assignment^b^	Dev./kHz	Unc./kHz	Ref.	Comment^d^^,^^e^
2.160	**2 160**037.3 ± 18.3	(0 1 0)4_2,2_ ← (0 1 0)5_1,5_	–57.3	300	(51)	P
12.009	12 008 811.5 ± 13.0	(0 1 0)4_2,3_ ← (0 1 0)3_3,0_	–11.5	30	(52)	P
22.235	*22 235 079*.85 ± 0.05	(0 0 0)6_1,6_ ← (0 0 0)5_2,3_	0	0.05	(25)	O^7^
67.804	67 803 952.4 ± 16.8	(0 1 0)4_1,4_ ← (0 1 0)3_2,1_	7.6	40	(52)	P
96.261	**96 261**169.6 ± 22.8	(0 1 0)4_4,0_ ← (0 1 0)5_3,3_	–9.6	100	(52)	O^64^
119.996	119 995 933.5 ± 18.1	(0 1 0)2_2,0_ ← (0 1 0)3_1,3_	6.5	100	(52)	P
183.310	*183 310 087*.0 ± 1.0	(0 0 0)3_1,3_ ← (0 0 0)2_2,0_	0	1	(26)	O^65^
209.118	**209 118**548.9 ± 27.3	(0 1 0)5_5,1_ ← (0 1 0)6_4,2_	–178.9	100	(56)	P
232.687	**232 686**739.5 ± 17.5	(0 1 0)5_5,0_ ← (0 1 0)6_4,3_	–39.5	50	(54)	O^64^
293.664	293 664 476.0* ± 27.5	(0 1 0)6_6,1_ ← (0 1 0)7_5,2_	–34.0	100	(56)	O^66^
321.226	*321 225 677*.0 ± 0.6	(0 0 0)10_2,9_ ← (0 0 0)9_3,6_	0	0.6	(27)	O^67^
325.153	*325 152 899*.0 ± 1.0	(0 0 0)5_1,5_ ← (0 0 0)4_2,2_	0	1	(26)	O^68^
336.228	336 227 905.8 ± 19.8	(0 1 0)5_2,3_ ← (0 1 0)6_1,6_	35.2	50	(61)	O^69^
380.197	*380 197 359*.8 ± 0.1	(0 0 0)4_1,4_ ← (0 0 0)3_2,1_	0	0.1	(27)	O^70^
437.340	*437 346 664*.0 ± 2.0	(0 0 0)7_5,3_ ← (0 0 0)6_6,0_	0	2	(26)	O^71^
439.151	*439 150 794*.8 ± 0.3	(0 0 0)6_4,3_ ← (0 0 0)5_5,0_	0	0.3	(27)	O^71^
443.020	*443 018 354*.6 ± 0.8	(0 0 0)7_5,2_ ← (0 0 0)6_6,1_	0	0.8	(27)	O^72^
448.001	*448 001 077*.5 ± 0.5	(0 0 0)4_2,3_ ← (0 0 0)3_3,0_	0	0.5	(27)	P(abs)
470.890	*470 888 903*.0 ± 2.0	(0 0 0)6_4,2_ ← (0 0 0)5_5,1_	0	2	(26)	O^71^
474.689	*474 689 108*.0 ± 1.0	(0 0 0)5_3,3_ ← (0 0 0)4_4,0_	0	1	(26)	O^72^
488.491	*488 491 128*.0 ± 3.0	(0 0 0)6_2,4_ ← (0 0 0)7_1,7_	0	3	(26)	P
546.691	**546 690**528.3 ± 18.8	(0 1 0)5_2,4_ ← (0 1 0)4_3,1_	–9.3	20	(61)	P
620.701	*620 700 954*.9 ± 0.6	(0 0 0)5_3,2_ ← (0 0 0)4_4,1_	0	0.6	(27)	O(abs)^73^
658.007	658 006 361.0 ± 9.5	(0 1 0)1_1,0_ ← (0 1 0)1_0,1_	139.0	30	(53)	O^74^
899.302	899 302 020.1 ± 14.2	(0 1 0)2_0,2_ ← (0 1 0)1_1,1_	102.9	30	(61)	P
902.609	902 609 435.1 ± 12.6	(0 1 0)3_1,2_ ← (0 1 0)2_2,1_	75.9	30	(61)	P
916.172	**916 171**449.9 ± 8.3	(0 0 0)4_2,2_ ← (0 0 0)3_3,1_	–44.9	13	(57)	P(abs)
923.113	923 113 296.7 ± 10.1	(0 1 0)6_2,5_ ← (0 1 0)5_3,2_	48.3	30	(61)	P
968.047	968 046 960.7 ± 13.6	(0 1 0)8_2,7_ ← (0 1 0)7_3,4_	–2.7	50	(61)	P
970.315	970 315 045.1 ± 9.4	(0 0 0)5_2,4_ ← (0 0 0)4_3,1_	–77.1	18	(57)	O^75,76^
1 077.763	1 077 762 980.6* ± 21.5	(0 1 0)7_2,6_ ← (0 1 0)6_3,3_	59.4	50	(61)	P
1 153.127	1 153 126 820.2 ± 6.3	(0 0 0)3_1,2_ ← (0 0 0)2_2,1_	1.8	13	(57)	P
1 158.324	1 158 323 846.3 ± 7.3	(0 0 0)6_3,4_ ← (0 0 0)5_4,1_	–103.3	25	(57)	P
1 172.526	1 172 525 840.3 ± 9.0	(0 0 0)7_4,4_ ← (0 0 0)6_5,1_	–9.3	50	(61)	P
1 205.789	1 205 789 113.8 ± 11.4	(0 1 0)1_1,1_ ← (0 1 0)0_0,0_	–18.8	75	(61)	P
1 278.266	**1 278 265**917.1 ± 9.8	(0 0 0)7_4,3_ ← (0 0 0)6_5,2_	28.9	20	(57)	P
1 296.411	1 296 411 048.5 ± 9.6	(0 0 0)8_2,7_ ← (0 0 0)7_3,4_	–15.5	13	(57)	P
1 322.065	1 322 064 738.5 ± 5.4	(0 0 0)6_2,5_ ← (0 0 0)5_3,2_	64.5	13	(57)	P
1 344.676	1 344 676 162.9 ± 9.1	(0 0 0)7_4,4_ ← (0 0 0)8_1,7_	–2.9	40	(61)	P
1 440.782	**1 440 781**685.0 ± 9.0	(0 0 0)7_2,6_ ← (0 0 0)6_3,3_	–16.0	20	(61)	P
1 494.058	**1 494 057**515.5 ± 19.6	(0 1 0)2_2,0_ ← (0 1 0)2_1,1_	26.5	50	(61)	P
1 541.967	1 541 967 020.0 ± 9.7	(0 0 0)6_3,3_ ← (0 0 0)5_4,2_	–235.0	23	(57)	P
1 574.232	1 574 232 157.2 ± 7.7	(0 0 0)6_4,3_ ← (0 0 0)7_1,6_	–84.2	200	(59)	P
1 643.919	1 643 919 169.9 ± 13.8	(0 1 0)3_0,3_ ← (0 1 0)2_1,2_	219.1	42	(60)	P
1 740.398	**1 740 398**139.6 ± 19.3	(0 1 0)8_3,6_ ← (0 1 0)7_4,3_	–46.3	50	(62)	P
1 753.916	1 753 915 569.1 ± 12.5	(0 1 0)2_1,2_ ← (0 1 0)1_0,1_	–30.1	50	(62)	P
1 766.199	**1 766 198**689.0 ± 6.3	(0 0 0)7_3,5_ ← (0 0 0)6_4,2_	59.0	13	(57)	P
1 884.888	1 884 887 835.1 ± 8.4	(0 0 0)8_4,5_ ← (0 0 0)7_5,2_	–13.1	18	(57)	P

a Approximate (“rest”) frequencies obtained from ref (21).

b Recommended transition frequencies ± their uncertainties (1σ), derived in the present work and attached with their rovibrational assignments. The boldfaced predictions are based on newly observed Lamb-dip positions listed in Table 1. The two asterisked predictions involve “new” states [namely, (0 1 0)7_2,6_ and (0 1 0)7_5,2_], which are unknown from our previous SNAPS study.^32^

c Most accurate laboratory measurements from the literature. The column “Dev.” contains the deviations of the measured positions from the recommended values of this table. The column “Unc.” includes the uncertainties taken from the individual data sources. The italicized frequencies of the second column coincide with the measured literature values, leading to zero deviations.

d Short comments: “O” means an observed maser line reported in the cited reference, “P” is a predicted maser transition, and “(abs)” indicates that a maser line is not (easily) observable due to strong terrestrial absorption.

e The 380.197 GHz line was listed as a predicted maser line by Gray et al.,^21^ but in fact it has been observed in ref (70).

Looking at Table 2, it becomes clear that the uncertainties of the SNAPS-based recommended frequencies fall below 30 kHz for H_2_^16^O. More specifically, the lines belonging to the (0 0 0) and (0 1 0) vibrational states span the 0.05–9.7 and 9.5–27.5 kHz accuracy ranges, respectively. The reason behind the larger uncertainties of the 1 ⇐ 1 frequencies is that (a) their best paths are typically longer than those of the 0 ⇐ 0 lines, and (b) the 5 ⇐ 1 transitions demanded for the 1 ⇐ 1 predictions were recorded at higher (usually 0.25 Pa) pressure values due to their increased line widths, leading to elevated total uncertainties for the 5 ⇐ 1 Lamb dips. Of the SNAPS-based frequencies, 13 benefit from the new transitions presented in Table 1. In another 13 cases, typeset in italics, our SNAPS predictions coincide with those measured in refs (25−27) at the (sub-)kHz level; thus, no further improvement could be carried out in this study for them. It must be stressed, however, that these 13 highly accurate frequencies are confirmed, within 10–15 kHz, via network cycles (see, for example, the cycle displayed in Figure 2c, involving two new Lamb-dip lines).

Table 2 also provides a comparison with the most accurate previous laboratory measurements (see columns 4–6). Except for the 13 cases with very low (<3 kHz) uncertainties, the SNAPS approach delivers significantly more accurate frequencies: in several cases, the improvement reaches a factor of 10 over previous results. For a few maser transitions, large deviations are found, even above 100 kHz for six lines^53,56,57,60,61^ and outgrowing the uncertainty values by 4σ for three examples.^57,60^ In ref (52), Kuze reported overly conservative uncertainty estimates: the deviations remain well within 0.5σ for the four lines taken from it.

### 5.2 H_2_^18^O Lines

For the less abundant H_2_^18^O species, no maser action has been detected and the astronomical observations are mostly related to absorption lines among low-lying (0 0 0) rotational states. These transitions fall typically into the sub-mm range, outside the transmission window of the Earth’s atmosphere. As an application of the SNAPS approach to H_2_^18^O lines of astronomical interest, a sample of Herschel-based observations have been collected from the literature. Eleven of these transitions were probed with the PACS device in the luminous NGC 4418 and Arp 220 galaxies,^22^ while three via the HIFI instrument targeting the NGC 7129 star-forming region.^23^ These 14 transitions, plus 7 extra lines with boldfaced SNAPS frequencies, can be found in Table 3.

**Table 3:** Recommended Frequencies for Selected H_2_^18^O Lines of (Potential) Astronomical Relevance^a^

Line frequency			Laboratory measurements			
Rest/GHz	Recommended/kHz	Rovibrational assignment	Dev./kHz	Unc./kHz	Ref.	Comment
5.625	5 625 178.6* ± 7.9	(0 0 0)6_1,6_ ← (0 0 0)5_2,3_	–31.6	15	(77)	U[5.6 × 10^–29^]
467.089	467 088 662.3* ± 17.9	(0 0 0)10_3,7_ ← (0 0 0)11_2,10_	–52.3	500	(55)	U[7.8 × 10^–28^]
547.676	547 676 461.8 ± 6.2	(0 0 0)1_1,0_ ← (0 0 0)1_0,1_	8.2	15	(26)	O–I^23^
994.675	994 674 394.8 ± 5.6	(0 0 0)2_0,2_ ← (0 0 0)1_1,1_	36.2	36	(58)	O–I^23^
1 095.627	1 095 628 918.0 ± 5.4	(0 0 0)3_1,2_ ← (0 0 0)3_0,3_	37.0	36	(58)	O–I^23^
1 633.479	1 633 482 647.0 ± 5.5	(0 0 0)2_2,1_ ← (0 0 0)2_1,2_	3.0	36	(58)	O–II^22^
1 719.250	1 719 249 729.9 ± 5.6	(0 0 0)3_0,3_ ← (0 0 0)2_1,2_	–0.9	36	(58)	O–II^22^
2 147.726	2 147 731 662.5 ± 4.8	(0 0 0)3_1,3_ ← (0 0 0)2_0,2_	107.5	36	(58)	O–II^22^
2 622.948	2 622 939 652.5 ± 4.7	(0 0 0)4_1,4_ ← (0 0 0)3_0,3_	14.5	42	(58)	O–II^22^
2 741.661	2 741 672 239.7 ± 5.7	(0 0 0)2_2,1_ ← (0 0 0)1_1,0_	45.3	36	(58)	O–II^22^
3 296.741	3 296 734 323.1 ± 5.6	(0 0 0)3_2,2_ ← (0 0 0)2_1,1_	63.9	39	(58)	O–II^22^
3 636.466	3 636 466 222.6 ± 7.4	(0 0 0)6_1,6_ ← (0 0 0)5_0,5_	157.4	44	(58)	O–II^22^
3 696.249	3 696 249 155.3* ± 16.9	(0 0 0)9_4,6_ ← (0 0 0)9_3,7_	76.7	38	(58)	U[1.2 × 10^–23^]
3 769.504	3 769 506 438.8 ± 4.8	(0 0 0)4_2,3_ ← (0 0 0)3_1,2_	8.2	46	(58)	O–II^22^
3 951.553	3 951 581 619.1 ± 5.5	(0 0 0)3_2,1_ ← (0 0 0)2_1,2_	–441.8	899	(63)	O–II^22^
4 022.059	4 022 058 495.0* ± 11.4	(0 0 0)10_4,7_ ← (0 0 0)10_3,8_	153.0	277	(58)	U[1.4 × 10^–23^]
4 055.476	4 055 475 627.4* ± 12.4	(0 0 0)9_3,7_ ← (0 0 0)9_2,8_	–41.4	39	(58)	U[2.0 × 10^–23^]
4 416.311	4 416 284 119.9 ± 5.8	(0 0 0)3_3,1_ ← (0 0 0)2_2,0_	–25.9	44	(58)	O–II^22^
4 557.467	4 557 466 941.5* ± 9.8	(0 0 0)10_3,8_ ← (0 0 0)10_2,9_	–51.5	51	(58)	U[2.6 × 10^–23^]
4 785.166	4 785 166 359.1* ± 11.4	(0 0 0)9_2,8_ ← (0 0 0)9_1,9_	85.9	44	(58)	U[2.9 × 10^–23^]
5 051.263	5 051 272 429.1 ± 5.0	(0 0 0)4_3,2_ ← (0 0 0)3_2,1_	84.9	80	(58)	O–II^22^

a The columns carry the same meaning as in Table 2. In the last column, “O–I” and “O–II” indicate that a line was observed in astronomical environments I (NGC 7129) and II (NGC 4418/Arp 220), respectively, while “U” means that a transition has not yet been identified in astronomical sources. Comment “U” is always followed by the respective HITRAN intensity,^78^ multiplied by 0.002 (that is, the terrestrial relative abundance of H_2_^18^O) and given in cm molecule^–1^. The boldfaced (and asterisked) frequencies of the lines with comment “U” exploit the newly measured Lamb-dips presented in Table 1, involving new rotational states compared to ref (32).

Utilizing the best paths extracted from the ultraprecise H_2_^18^O network, accurate SNAPS predictions could be determined for the 21 transition frequencies of Table 3. In the second column of this table, the 14 “plain” frequencies, obtained for the lines observed by Herschel, are characterized by 5–8 kHz accuracy. The uncertainties of these SNAPS-based predictions are lower, in all cases, than those arising from direct measurements.^26,55,58,63^ Except for three cases, the deviations of the literature positions from our predicted frequencies are smaller than the 2σ uncertainty limits.

During the experimental campaign of the present work, it was noticed that there remained only six 0 ⇐ 0 transitions in the experimental data sets of Belov et al.^55^ and Matsushima et al.^58^ whose upper or lower states had not been connected to the ultraprecise H_2_^18^O network. This inspired us to record further Lamb-dip lines, given in Table 1, for H_2_^18^O. The six additional 0 ⇐ 0 transitions, along with a microwave line around 6 GHz, are listed in Table 3 with boldfaced SNAPS predictions. For the 6 GHz transition, which possesses the same assignment as the well-studied maser line of H_2_^16^O at 22 GHz (see Table 3), the frequency uncertainty could be halved via SNAPS, with respect to an old laboratory measurement.^77^ The boldfaced frequencies of Table 3, especially those with large attached intensities, may prove useful in future astronomical investigations.

## 6 Discussion and Conclusions

In the present study, the SNAPS method^24^ was utilized to obtain ultrahigh-precision frequency predictions for selected H_2_^16^O and H_2_^18^O transitions of astronomical significance. Ultraprecise H_2_^16^O and H_2_^18^O networks, lying at the heart of this investigation, were built with the help of new near-infrared Lamb-dip lines, observed using our second-generation NICE–OHMS spectrometer.^36^ The increased sensitivity of this upgraded NICE–OHMS setup enables the measurement of molecular transitions at a very low pressure (0.1 Pa or even less), thus decreasing the pressure shifts to an almost negligible amount. This stringent pressure condition, coupled with frequency-comb-based calibration, leads to kHz accuracy for the retrieved positions. Unfortunately, this is not the case for HD^16^O, another water isotopologue relevant in outer space, as the near-infrared Lamb dips of semiheavy water are significantly shifted/distorted during the NICE-OHMS measurements due to laser-induced Stark mixing, especially for min (*K*_*a*_^′^,*K*_*a*_^″^)>3.^79^

By measuring nearly 600 NICE–OHMS lines, chosen via the SNAPS protocol and combined with extremely accurate literature transitions,^25−29,39^ a large number of (0 0 0)^24,30^ and (0 1 0)^32^ states could be included in the ultraprecise H_2_^16^O and H_2_^18^O networks. For the exploration of the (0 0 0) rotational states, a single probe laser at 1.4 μm proved to be sufficient to form serial Λ schemes, whereby both lower states belong to (0 0 0).^24^ Nevertheless, to attain the (0 1 0) states from the *ortho*/*para* ground state, an extra laser, operating around 1.2 μm, had to be involved, ensuring the construction of Λ schemes where one of the lower states pertains to (0 0 0) and the other to (0 1 0).^32^ These design principles were kept in mind when the new Lamb-dip lines of the present work were selected for detection, closing most of them into network cycles to verify their internal consistency (see, e.g., the two green transitions shown in Figure 2c). This procedure led to accurate predictions for nine rotational frequencies, whose upper/lower states were not covered in our previous analysis.^32^

From the smallest-uncertainty paths of the ultraprecise H_2_^16^O and H_2_^18^O networks, predicted frequencies could be extracted, with a few kHz uncertainty, for a collection of 68 astronomical lines. This high accuracy was achieved because the predictions inherited, by design, the ultrahigh precision of the near-infrared NICE–OHMS transitions^24,30−32^ and other lines with (sub)-kHz accuracy.^25−27^ For somewhat floppy molecules such as water, the SNAPS method is clearly superior to an EH model, the usual representation of quantum states in high-resolution spectroscopy. The issues with EH models are even more pronounced for states in the highly excited *P* = 4 and *P* = 5 polyads, where there are strong interactions among closely spaced states of the same symmetry. For example, while there was an attempt to reach high accuracy via an EH fit for the *P* = 4 polyad of H_2_^16^O,^80^ the fitting error could not be decreased below 4 GHz, an unacceptably large value in light of the kHz-level uncertainties achieved by today’s precision-spectroscopy techniques.

It is truly remarkable that the high precision of near-infrared Lamb-dip spectroscopy could be transferred, via the SNAPS method, to astronomical transitions at a competitive level. Apart from 13 pure rotational lines, which are also included in the ultrahigh-accuracy H_2_^16^O network, our frequency predictions turned out to be more accurate than the direct microwave and sub-mm spectroscopy measurements (see Tables 2 and 3). In several cases, large deviations were found, up to 100–200 kHz, exceeding the claimed uncertainties^53,56,57,60,61^ even by 5σ and demonstrating the need for updated line positions. These considerable deviations may partly arise from the higher pressures employed in some of the data sources. For example, the transitions of Matsushima et al.^57^ were recorded at 4.7 Pa, whereas the lines behind our predictions were investigated at much lower pressures, 0.01–0.55 Pa. Taking a pressure slope of ±20 kHz Pa^–1^, an effective value ascertained for near-infrared Lamb dips,^24,30−32^ 4.7 Pa may be translated to a pressure shift of ±94 kHz, which is reasonably close to the 100 kHz level reflected by the problematic deviations.

Due to electric-dipole selection rules, the SNAPS protocol hinges on the inclusion of a few highly accurate rotational transitions in the network, needed to attach opposite-parity states within the same vibrational manifold. However, if quadrupole lines were available and combined with dipole transitions, such connections could be made without reliance on pure rotational lines. In fact, quadrupole transitions have been detected for H_2_^16^O in Doppler-broadened CRDS spectra with 60–90 MHz uncertainty.^81,82^ However, high-quality Lamb dips would be demanded for our purposes, such that measurement might be possible if suggested by the recent detection of a Lamb-dip feature probed for a quadrupole transition in the first overtone of H_2_, yielding a highly accurate position for this line.^36^ Alternatively, two-photon transitions could also be applied as direct links among clusters of one-photon lines with different-parity lower states. Double resonance techniques^83,84^ bear promise for intracavity observations of two-photon lines in water, securing the desired kHz accuracy.

Most of the purely rotational water lines derived in this study have immediate astronomical relevance: (a) 48 transitions of H_2_^16^O (may) act as masers in evolved-star envelopes,^21^ and (b) 14 lines of H_2_^18^O have been detected^22,23^ in extragalactic regions. The accurate frequencies determined for these transitions could be valuable in numerous applications, including the analysis of kinematics, Doppler motions, and redshifts in astronomical objects, as well as the investigation of inflows and outflows characterizing these celestial sources. Moreover, due to the omnipresence of water, the recommended frequencies of Tables 2 and 3 can be employed as reference values to calibrate new high-resolution spectra of astronomically relevant molecules in the 0–5 THz frequency region.

As to the applicability of the SNAPS method to other molecules, it is noted that our current NICE–OHMS setup is designed for probing stable closed-shell molecules. Similar optical technologies have been developed to probe molecular ions^85^ and open-shell molecular radicals,^86^ but the accuracy obtained is still insufficient to extract competitive frequencies in the radio domain. As an another molecule, acetylene has been investigated via cavity-enhanced techniques within a network approach,^87^ but this species may be less relevant from an astronomical perspective. A molecule suitable for future SNAPS studies is methanol, which exhibits important maser action on a multitude of lines in the Milky Way and in extragalactic sources.^88^ In the near-infrared region, there are numerous vibrational bands of methanol which could be subject to a SNAPS analysis for the extraction of ultraprecise radio-line frequencies.^89^ These accurate frequency predictions could play a decisive role in the quest for probing variation of fundamental constants, like the proton–electron mass ratio, on a cosmological time scale,^90,91^ as well as to test the weak equivalence principle.^92^

For the extensive study of starless cores,^93^ space-related fundamental physics,^91,92^ as well as hyperfine-resolved maser observations,^94^ the experimental resolution and accuracy of existing radio observatories is well suited. The upcoming upgrade of the ALMA observatory,^14^ allowing a unique spectral resolution of 1–30 kHz over the entire ALMA bandwidth, will open up new territories within the realm of astronomical spectroscopy. In this situation, the arrival of ultraprecise radio lines, such as those provided here for water, is well-timed to address the demands of contemporary astronomy.

## Supporting Information Available

Detailed descriptions of Supporting Information tables (PDF)
(a) Machine-readable list of new H_2_^16^O and H_2_^18^O transitions recorded with our upgraded NICE-OHMS setup and (b) detailed comparison of previous laboratory measurement results with the SNAPS-predicted frequencies obtained for the H_2_^16^O and H_2_^18^O lines of Tables 2 and 3, respectively (ZIP)
