# Supplementary material for: A Network Approach for the Accurate Characterization of Water Lines Observable in Astronomical Masers and Extragalactic Environments
Source: ACS Earth Space Chem. 2024 Aug 9;8(9):1901–12. doi: 10.1021/acsearthspacechem.4c00161 (PMC11417992; doi:10.1021/acsearthspacechem.4c00161)
Supplement: Supplementary file 1 — sp4c00161_si_001.pdf [file sp4c00161_si_001.pdf]

Supporting Information for the paper

# "A network approach for the accurate characterization of water lines observable in astronomical masers and extragalactic environments"

by

Wim Ubachs [1*], Attila G. Császár [2-3], Meissa L. Diouf [1], Frank M. J. Cozijn [1],

and Roland Tóbiás [2-3*]

[1] Department of Physics and Astronomy, LaserLaB, Vrije Universiteit,

De Boelelaan 1081, 1081 HV Amsterdam, The Netherlands

[2] Institute of  Chemistry, ELTE Eötvös Loránd University,

H-1518 Budapest 112, P.O. Box 32, Hungary

[3] HUN-REN-ELTE Complex Chemical Systems Research Group,

H-1117 Budapest, Pázmány Péter sétány 1/A, Hungary

[*] Corresponding authors:

- W. Ubachs [email: w.m.g.ubachs@vu.nl ]
- R. Tóbiás [email: roland.tobias@ttk.elte.hu]

Four files:

table_S1.txt  :
List of new experimental H2O-16 transitions recorded with the NICE-OHMS technique

table_S2.txt  :
List of new experimental H2O-18 transitions recorded with the NICE-OHMS technique

table_S3.txt  :
Detailed comparison of previous laboratory measurement results with the SNAPS-predicted frequencies obtained for the H2O-16 lines of Table 2 in the main text

table_S4.txt  : Detailed comparison of previous laboratory measurement results with the SNAPS-predicted frequencies obtained for the H2O-18 lines of Table 3 in the main text
